# MIP-3-Alpha and MIP-3-Beta as Early Predictors of Pneumonia in Polytraumatized Patients

**DOI:** 10.1007/s00408-025-00799-2

**Published:** 2025-03-13

**Authors:** Gregor Wollner, Florian Hruska, Paul Ettel, Thomas Weichhart, Felix R. M. Koenig, Lukas L. Negrin

**Affiliations:** 1 Department of Orthopedics and Trauma-Surgery, Medical University of Vienna, 1090 Vienna, Austria; 2 Department of Biomedical Imaging and Image-Guided Therapy, Medical University of Vienna, Vienna, Austria; 3 Center for Pathobiochemistry and Genetics, Medical University of Vienna, Vienna, Austria

**Keywords:** Polytrauma, Pneumonia, Biomarker, MIP-3-alpha, CCL20, MIP-3-beta, CCL19, Serum levels

## Abstract

**Introduction:**

Pneumonia is one of the most common complications in patients suffering multiple traumas and is associated with an exceptionally high mortality rate. MIP-3-alpha and MIP-3-beta are pro-inflammatory chemokines expressed in the pulmonary mucosa and are reported to play a crucial role in inflammation. Thus, the present study aimed to investigate whether there is an association between MIP-3-alpha- and MIP-3-beta expression and manifestation of pneumonia in patients suffering polytrauma.

**Material and Methods:**

This prospective outcome study was conducted at our level I trauma center, and 110 polytraumatized patients (Injury Severity Score ≥ 16, ≥ 2 body regions) were prospectively enrolled (median age, 39 years; median Injury Severity Score (ISS), 33; 70.9% male) over four years. Protein levels were assessed at admission (day 0) and subsequently on days 1, 3, 5, 7, and 10 during routine blood draws, utilizing one separation gel tube for each measurement. Furthermore, the correlation between MIP-3-alpha- and MIP-3-beta expression and the manifestation of pneumonia was calculated.

**Results:**

We observed significantly higher levels of MIP-3-beta expression over the entire time course in the pneumonia cohort. MIP-3-alpha levels were elevated on days 3, 5, 7, and 10 post-trauma in patients suffering from pneumonia. In contrast, no comparable pattern was observed for other pro- and anti-inflammatory cytokines (*e.g.,* IL-6 or TNF-alpha). A peak of serum level expression was documented on day 5 in both biomarkers (MIP-3-alpha 51.8 pg/mL; MIP-3-beta 328.0 pg/mL). ROC analysis provided a cut-off value of 19.3 pg/mL (sensitivity 0.87, specificity 0.33; AUC 0.757) for MIP-3-alpha, whereas a cut-off value of 209.5 pg/mL (sensitivity 0.78, specificity 0.34; AUC 0.757) was determined for MIP-3-beta on day 5.

**Conclusion:**

The present study demonstrated elevated MIP-3-alpha and MIP-3-beta levels as sensitive pneumonia predictors in patients with multiple traumas. These biomarkers allow for identifying patients at high risk of developing pneumonia at an early stage.

## Introduction

Trauma is the primary cause of death in the first world for patients between the ages of one and forty-five, accounting for around six million fatalities annually globally [1]. Pneumonia is one of the most common complications in patients suffering multiple trauma and is associated with an exceptionally high mortality rate [2–4]. Pneumonia is an infectious process involving the lung parenchyma defined by an infiltration of pathogens leading to exudative fluid and migration of inflammatory cells in the pulmonary tissue, resulting in impaired respiratory function [5]. From 27% up to 44% of all patients suffering multiple traumas develop pneumonia, which is known to be four times higher compared to the non-trauma intensive care unit (ICU) population [2, 3, 6, 7]. This makes pneumonia one of the most common complications after trauma [8]. It is associated with higher mortality rates and extended durations of hospitalization, as well as prolonged stays at the ICU in polytraumatized patients [2, 9]. Furthermore, trauma patients suffering from pneumonia are more likely to require rehabilitation or admission to a skilled nursing facility, making it a socioeconomic challenge as well [2, 10]. While it is widely acknowledged that the most significant risk factor for the development of pneumonia is mechanical breathing, patients with previous trauma seem to be especially vulnerable [2, 4]. Traumatic brain injury (TBI), chest trauma, spinal cord injury (SCI), an Injury Severity Score (ISS) > 25, the presence of a lung contusion, and undergoing laparotomy are trauma-associated risk factors for developing pneumonia [2, 11]. Apart from trauma, multiple other risk factors have been described, such as age, male gender, patient positioning, cardiac disease, COPD, DM II, immunosuppression, and intubation [2, 12, 13].

After the initial trauma, a cascade of prolonged systemic inflammation frequently results in the manifestation of sepsis, acute respiratory distress syndrome (ARDS), or pneumonia [1, 4, 14]. Cytokines and chemokines have been identified as major components in the regulation and dysregulation of the inflammatory response to traumatic injuries [1, 14, 15].

In particular, cytokines such as IL-4, IL-6, IL-7, IL-8, IL-10, and TNF-alpha are key signaling molecules within the immune system, secreted by various immune and non-immune cells [16]. They are classified into pro- and anti-inflammatory properties [17]. Within the first hours post-trauma, pro-inflammatory cytokines activate—among others—the phagocytic activity of leucocytes, which serve as the primary line of immune defense [16].

Chemokines, on the other side, regulate leukocyte chemotaxis [18, 19]. Chemokines are low-molecular weight (8–14 kDa) proteins that are categorized based on structural changes into C–C motif (CC), C-X-C motif (CXC), C motif (C), and CXC3 chemokines [19]. As of now, approximately 50 distinct types of chemokines have been recognized [19, 20]. An unbiased screening approach of multiple biomarkers in patients following trauma identified MIP-3-alpha, also known as Chemokine (C–C motif) ligand 20 (CCL20) and MIP-3-beta (C–C motif chemokine ligand 19, CCL19), as chemokines of special interest in the present study [19, 21]. It is suggested that both chemokines (MIP-3-alpha, MIP-3-beta) play a crucial role in developing sepsis and pneumonia, yet only little is known about their potential role in polytraumatized patients. Due to the expression of MIP-3-alpha and MIP-3-beta in the lung mucosa [21, 22], we hypothesize there is a possible correlation between these biomarkers and the development of pneumonia in patients suffering multiple traumas. Thus, the present study aimed to investigate whether MIP-3-alpha/beta expression is associated with the manifestation of pneumonia in patients suffering from polytrauma. The secondary objectives included evaluating further clinical differences between these two cohorts.

## Material and Methods

### Patients and Study Design

The present study received authorization from the Ethics Committee of the Medical University of Vienna (EK#1617/2018; August 28, 2018) and adhered to the protocols established by the Declaration of Helsinki. We recruited 110 patients from 09/2018 to 08/2022 adhering to the following inclusion criteria: a minimum age of 18 years, injuries ≥ 2 different body regions according to Abbreviated Injury Score (AIS), Injury Severity Score (ISS) of 16 or greater and duration of stay in the intensive care unit (ICU) for no less than one night. Patients suffering from malignant diseases, burn victims, and patients suffering from lung disorders were excluded. The following signs led to the diagnosis of pneumonia: body temperature over 38 °C or under 35.5 °C, leukocytosis (white cell count > 10,000/mm^3^) or leucopenia (white cell count < 4000/mm^3^), macroscopically purulent sputum, dyspnea, tachypnea, or a new occurred cough in spontaneous breathing patients as well as radiographic sign, such as a chest infiltrate. Furthermore, bronchoalveolar lavage (BAL) was performed in all of the intubated patients, and the diagnosis of pneumonia was confirmed if it contained more than 105 organisms/ml. In spontaneously breathing patients induced sputum was obtained and diagnosis of pneumonia was based on microbiological testing in a similar manner.

### Evaluation of Serum Levels

During routine blood withdrawal, blood specimens were obtained from each polytraumatized individual during the initial clinical assessment upon admission and subsequently on days 1, 3, 5, 7, and 10 utilizing a single separation gel tube (Vacuette R© 8 mL; Greiner Bio-One International; Austria). Blood samples were consecutively centrifuged at 3000 × *g* for 15 min to separate the serum. Subsequently, the samples were stored at − 80 °C until analysis was performed. Biomarkers evaluated were MIP-3-alpha, MIP-3-beta, IL-4, IL-6, IL-7, IL-8, IL-10, and TNF-alpha. The analysis was conducted utilizing the 7-Plex Luminex® Performance Human XL Cytokine Panel (Catalog no.: F CSTM18B-07; R&D Systems; Minneapolis, MN, USA) in conjunction with the Luminex® 200TM Instrument (Catalog no.: LX200-XPON-RUO; R&D Systems; Minneapolis, MN, USA). All measurements were conducted in duplicate, and mean values were calculated.

### Statistical Analysis

All statistical analyses were performed using the SPSS® 29 software (SPSS Inc., Chicago, IL).

The Kolmogorov–Smirnov test was performed to assess the normal distribution of the parameters. Normally distributed parameters were displayed as mean ± standard deviation, whereas non-normally distributed parameters are presented as median and interquartile range (IQR) in round brackets. Mann–Whitney *U* tests were performed to assess the differences between independent groups, while Wilcoxon signed-rank tests were utilized to evaluate variations in protein levels within a patient across multiple time points. Spearman’s correlation was calculated to display associations between protein levels. Multivariate logistic regression was calculated to assess association of pneumonia and selected parameters. Finally, receiver operating characteristic (ROC) was calculated. Youden’s Index was utilized to evaluate the cut-off value. Frequency counts and percentages characterize categorical data. They were analyzed using the *χ*^2^ test. Fisher’s exact test was applied when the sample size was small. Boxplots were established to visualize the results. In general, a *p* value < 0.05 was considered significant.

## Results

### Study Population

Our study group consisted of 110 polytraumatized patients with a median age of 39 years and a median ISS of 33. Seventy-eight (70.9%) patients in the present study were male gender. The study population in detail and the two cohorts’ injury-related data are expressed in Table 1. The pneumonia cohort consisted of 25 patients on admission. Due to a documented fatality as well as a lack of blood samples, the number of patients was consecutively reduced to 24 on days 1 and 3, 23 patients on days 5 and 7, and 22 patients on day 10. In the no pneumonia cohort, fatality as well as a lack of blood samples on day 1 led to the following distribution: 83 patients were documented on the day of admission, 75 patients on day 1, 77 patients on day 3, 69 patients on day 5, 66 patients on day 7, and 59 patients on day 10. In the present study, pneumonia was not associated with a higher mortality rate. Interestingly, mortality was more frequently observed in the non-pneumonia group, even though results were not significant. Injuries of the head and face resulted in significantly higher observations of pneumonia, whereas previous chest trauma did not show any differences in developing pneumonia within the two cohorts. Furthermore, a significant proportion of our patients experienced substantial injuries, as evidenced by ISS values that spanned from 17 to 66. However, higher ISS values were not associated with the manifestation of pneumonia in the present study. Duration of intubation and length of stay at the ICU and hospital were significantly higher in the pneumonia cohort. Furthermore, sepsis and ARDS were more frequently observed in the pneumonia cohort. There was no significant association between performed surgeries and the manifestation of pneumonia. The mean time of diagnosis of pneumonia was 6.3 days following trauma. *Staphylococcus aureus*-associated pneumonia was documented most frequently, with 9 (36%) observed cases, followed by *Enterobacter cloacae* and *Klebsiella pneumoniae*, each identified in 4 (16%) patients, *Pseudomonas aeruginosa* was detected in 3 (12%) cases. Multiple organisms have been identified in 20 (80%) patients. Furthermore, *Candida albicans* was observed in 16 (64%) cases—in all patients below the diagnostic threshold.

**Table 1:** Demographic and injury-related data of the two cohorts

	Pneumonia	No pneumonia	*p* Value
Number (*n*)	25	85	–
Age (years), median (IQR)	45 [18; 84]	35 [18; 91]	0.246
Males:females (n)	19:6	59:26	0.524
ISS, median	34 [17; 66]	30 [17; 59]	0.064
AIS			
Head, median	4 [0; 5]	2 [0; 5]	**0.006**
Thorax, median	3 [0; 5]	3 [0; 5]	0.170
Abdomen, median	0 [0; 5]	2 [0; 5]	0.262
Face, median	2 [0; 4]	0 [0; 3]	**0.026**
Extremities, median	3 [0; 4]	3 [0; 5]	0.944
External, median	1 [0; 2]	1 [0; 5]	0.075
Diagnosis of Pneumonia (days), mean ± SD	6.3 ± 4.5	–	–
Duration of Intubation (days), median (IQR)	19 [0; 61]	2 [0; 75]	**< 0.001**
Length of stay at the ICU (days), median (IQR)	31 [5; 145]	5 [0; 80]	**< 0.001**
Length of hospital stay (days), median (IQR)	71 [19; 206]	29 [0; 263]	**< 0.001**
Intubation at admittance (%)	17 (68.0)	45 (52.9)	0.182
Mortality (%)	1 (4.0)	18 (21.2)	0.068°
Injury causes			0.084°
Pedestrian hit by vehicle (%)	6 (24.0)	6 (7.4)	
Fall from height (%)	6 (24.0)	37 (45.7)	
Traffic accident (%)	9 (36.0)	27 (33.3)	
Hit by oncoming tram/subway (%)	3 (12.0)	11 (13.6)	
Hit by fallen tree branch (%)	1 (4.0)	0 (0)	
Surgeries			0.195
0 (%)	4 (16.0)	16 (18.9)	
1 (%)	3 (12.0)	27 (31.2)	
2 (%)	6 (24.0)	15 (17.7)	
3+ (%)	12 (48.0)	27 (31.8)	
Spine injury (%)	13 (52.0)	57 (69.5)	0.107
Myelopathy (%)	2 (8.0)	5 (6.3)	0.670°
Suicide attempt (%)	5 (20.0)	31 (36.9)	0.115
Sepsis (%)	7 (28.0)	8 (9.4)	**0.040°**
ARDS (%)	6 (24.0)	3 (3.5)	**0.004°**
Acute kidney failure (%)	5 (20.0)	10 (11.8)	0.325
Urinary tract infection (%)	9 (36.0)	8 (9.4)	**0.003**
Pancreatitis (%)	2 (8.0)	1 (1.2)	0.129

Bold indicates *p* < 0.05 and the small superscript circle indicates application of Fisher’s exact test

ISS, injury severity score; AIS, abbreviated injury scale; ARDS, acute respiratory distress syndrome; ICU, intensive care unit

There was no significant correlation between age or sex and manifestation of pneumonia. Multivariate logistic regression was calculated using the covariates age, gender, and ISS. Age (*p* = 0.232), gender (*p* = 0.524), and ISS (*p* = 0.051) did not show a significant association with pneumonia.

### MIP-3-Alpha-Expression

**Fig. 1:**
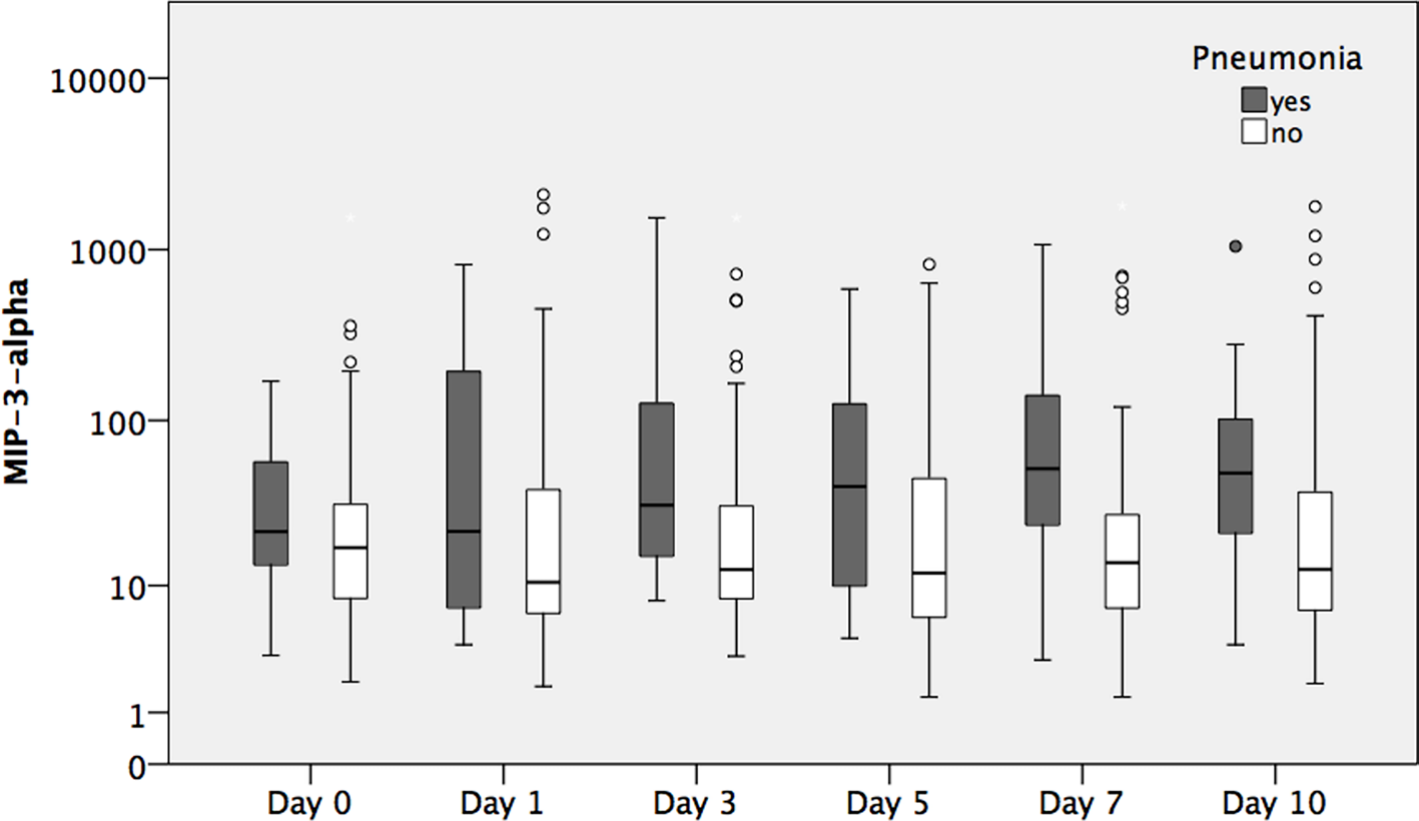
MIP-3-alpha expression over time course displayed in a logarithmic scale

Table 2 displays MIP-3-alpha expression over time course. We documented significantly higher expression of MIP-3-alpha in patients suffering pneumonia on day 3 (*p* = 0.017), day 5 (*p* < 0.001), day 7 *(p* < 0.003) and day 10 *(p* < 0.004). The peak of MIP-3-alpha expression was documented on day 5 with 51.8 pg/mL. The day of admission and day 1 did not show any significant correlations between the occurrence of pneumonia and MIP-3-alpha expression. Figure 1 displays MIP-3-alpha expression in pneumonia versus no pneumonia cohort over time.

**Table 2:** Display of selected proteins (in pg/mL) expression over time course. Displayed values are median [minimum, maximum]

	Day 0	Day 1	Day 3	Day 5	Day 7	Day 10
	*MIP-3-alpha*					
Pneumonia	21.68 [3.3; 170.0]	22.1 [4.0; 818.0]	40.7 [4.4; 588.4]	51.8 [3.1; 1069.4]	48.8 [4.0; 1044.7]	31.4 [8.0; 1532.8]
No pneumonia	17.3 [2.0; 1532.0]	10.5 [1.8; 2092.0]	12.0 [1.5; 820.4]	14.0 [1.5; 1793.0]	12.7 [2.0; 1781.7]	12.6 [3.3; 1527.3]
*p* value	0.227	0.129	**0.017**	**< 0.001**	**0.003**	**0.004**
	*MIP-3-beta*					
Pneumonia	79.4 [33.7; 591.0]	135.0 [38.0; 878.0]	318.5 [106.0; 724.0]	328.0 [86.6; 682.8]	246.0 [106.8; 684.0]	168.0 [55.4; 952.0]
No pneumonia	70.2 [15.9; 368.0]	80.1 [8.5; 772.0]	162.5 [17.6; 1104.6]	159.2 [39.0; 1512.6]	142.8 [40.3; 3910.4]	110.4 [51.1; 1159.2]
*p*-value	**0.036**	**0.040**	**0.001**	** < 0.001**	**0.001**	**0.005**
	*IL-4*					
Pneumonia	22.3 [4.0; 46.1]	16.2 [3.3; 44.5]	14.7 [3.3; 37.4]	13.3 [3.0; 40.4]	8.5 [3.0; 43.3]	11.1 [3.5; 41.3]
No pneumonia	19.1 [3.0; 98.9]	18.5 [1.8; 49.8]	15.6 [1.8; 49.8]	14.8 [3.0; 90.9]	13.5 [1.8; 12.3]	10.7 [2.2; 48.5]
*p*-value	0.535	0.815	1.000	0.212	0.328	0.744
	*IL-6*					
Pneumonia	95.8 [3.2; 2199]	79.9 [9.1; 589.0]	42.4 [7.2; 967.7]	28.2 [4.9; 664.4]	16.6 [3.4; 213.5]	12.1 [3.8; 262.4]
No pneumonia	92.0 [3.1; 21,671.9]	49.7 [5.4; 6873.1]	16.1 [0.58; 1716.2]	13.6 [0.5; 416.4]	10.0 [0.78; 669.3]	8.6 [1.1; 628.7]
*p*-value	0.841	0.826	**0.013**	**0.011**	0.415	0.111
	*IL-7*					
Pneumonia	8.2 [1.5; 17.1]	8.0 [1.3; 17.6]	8.4 [3.1; 17.1]	10.4 [2.1; 25.5]	11.0 [3.0; 40.2]	17.5 [3.7; 63.4]
No pneumonia	8.0 [1.2; 19.9]	7.1 [0.6; 19.2]	7.3 [0.8; 21.5]	9.4 [1.3; 26.6]	12.8 [3.5; 31.1]	16.1 [2.6; 46.5]
*p*-value	0.994	0.519	0.200	0.306	0.267	0.577
	*IL-8*					
Pneumonia	60.5 [13.3; 921.8]	43.0 [2.8; 816.1]	31.9 [5.9; 311.1]	36.2 [4.4; 474.5]	34.0 [8.6; 347.7]	39.5 [3.8; 1209.4]
No pneumonia	53.4 [11.7; 15,897.0]	44.8 [11.6; 4685.3]	34.2 [5.7; 557.7]	38.3 [9.7; 1301.6]	32.4 [10.2; 34,118.0]	32.7 [11.0; 1763.4]
*p*-value	0.575	0.707	0.528	0.702	0.431	0.408
	*IL-10*					
Pneumonia	25.4 [2.5; 273.8]	3.0 [0.77; 38.9]	1.6 [0.3; 19.2]	1.5 [0.44; 7.7]	1.2 [0.4; 7.1]	1.3 [0.6; 19.8]
No pneumonia	29.6 [0.1; 504.4]	3.4 [< 0.1; 92.3]	1.3 [0.1; 18.7]	1.1 [0.1; 13.9]	1.2 [< 0.1; 17.5]	1.2 [< 0.1; 7.7]
*p*-value	0.968	0.922	0.151	0.087	0.378	0.230
	*TNF-alpha*					
Pneumonia	5.2 [1.2; 12.9]	6.7 [1.8; 16.1]	8.0 [2.1; 13.1]	8.6 [1.9; 12.4]	6.7 [1.8; 15.6]	7.0 [1.9; 17.2]
No pneumonia	4.9 [0.1; 130.9]	4.8 [0.3; 24.1]	5.5 [1.6; 42.4]	6.2 [0.4; 53.8]	5.3 [0.4; 44.2]	5.3 [0.6; 50.2]
*p*-value	0.388	0.182	0.115	0.076	0.273	0.177

Bold indicates *p* < 0.05

### MIP-3-Beta-Expression

**Fig. 2:**
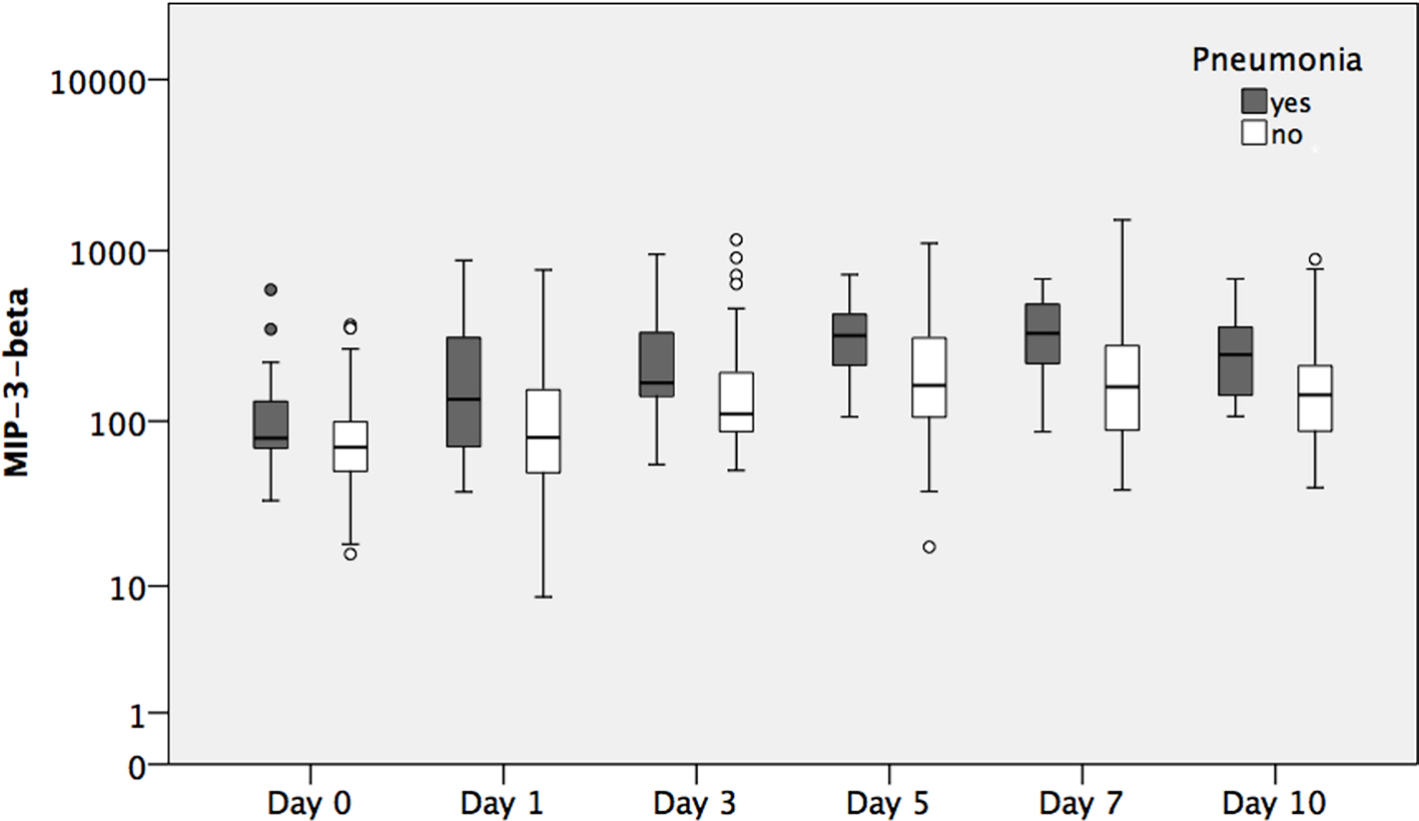
MIP-3-beta expression over time course displayed in a logarithmic scale

Table 2 shows MIP-3-beta expression over ten days. As seen in Fig. 2, MIP-3-beta expression is significantly higher over ten days in the pneumonia cohort. A peak of MIP-3-beta expression was also documented on day 5 with 328.0 pg/mL.

### Interleukin Expression

Furthermore, multiple cytokines, which have been implicated in regulating pneumonia pathophysiology, were assessed to evaluate if MIP-3-beta and -alpha evaluation were part of a systemic increase in cytokines. As seen in Table 2, IL-4, IL-6, IL-8, and IL-10 levels continuously decreased over time, whereas IL-7 showed an increase following trauma. However, except for IL-6, which was slightly decreased on days 3 and 5 in pneumonia patients, there was no difference between the groups.

### Spearman Correlations

Spearman’s correlation coefficients were calculated to evaluate the correlation between MIP-3-alpha and MIP-3-beta-expression over time. There was a significant positive correlation between the biomarkers on all selected dates (day 0: *r* = 0.456, *p* < 0.001; day 1: *r* = 0.501, *p* < 0.001; day 3: *r* = 0.742, *p* < 0.001; day 5: *r* = 0.752, *p* < 0.001; day 7: *r* = 0.628, *p* < 0.001; and day 10: *r* = 0.635, *p* < 0.001).

### ROC Statistics

**Fig. 3:**
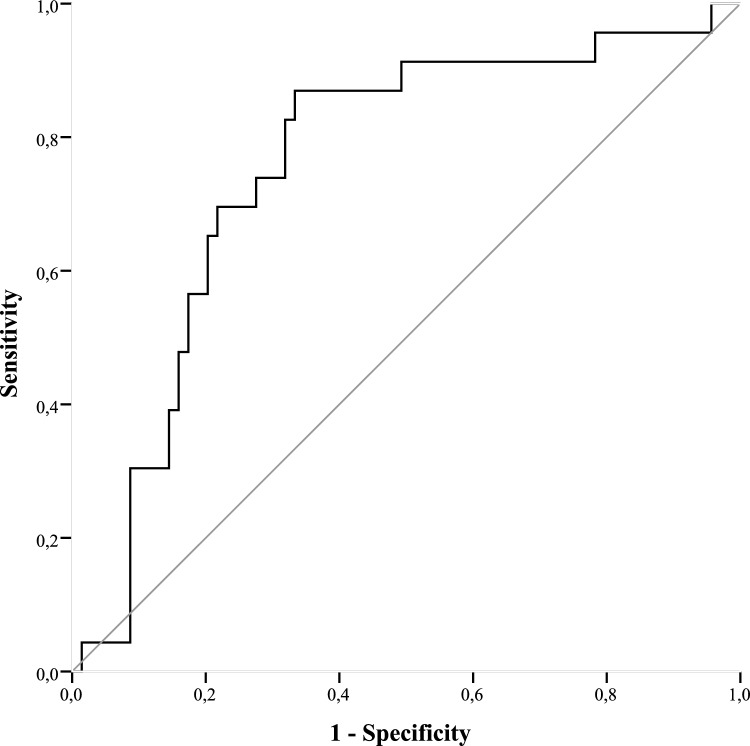
Receiver operating characteristic of MIP-3-alpha expression on day 5

**Fig. 4:**
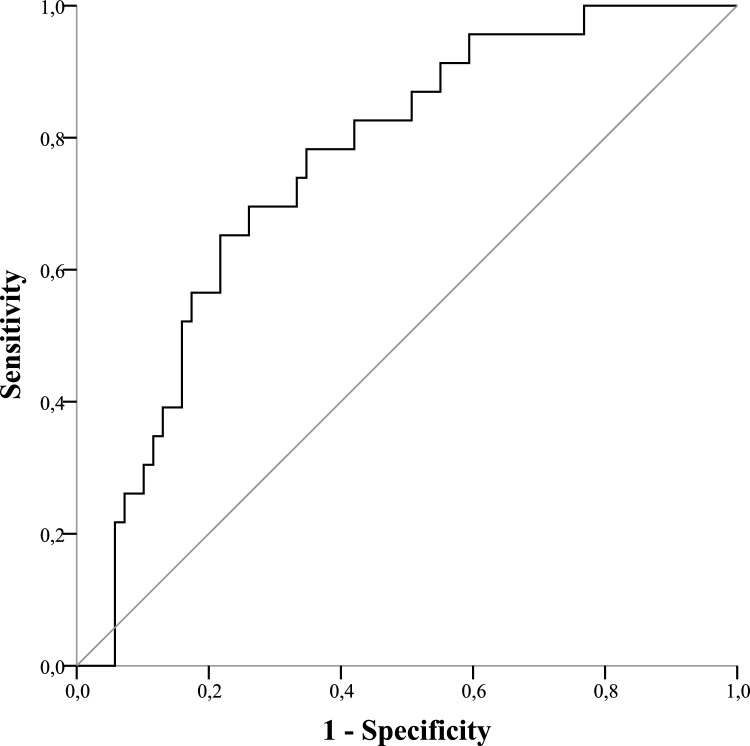
Receiver operating characteristic of MIP-3-beta expression on day 5

Days 0, 1, and 3 did not show sufficient results when calculating the area under the curve (AUC). As displayed in Fig. 3, the plotted ROC curve of MIP-3-alpha expression on day 5 and the manifestation of pneumonia showed an AUC of 75.7% (sensitivity 0.87, specificity 0.33) and a cut-off value of 19.3 pg/mL. Figure 4 displays an AUC of MIP-3-beta expression of 75.7% (sensitivity 0.78, specificity 0.34) and a cut-off value of 209.5 pg/mL was evaluated.

## Discussion

The primary discovery of this study is that polytraumatized patients suffering from pneumonia during hospitalization have been shown to express MIP-3-alpha and MIP-3-beta at higher levels compared to patients where pneumonia was absent. The disparities observed were statistically significant on days 3, 5, 7, and 10 in MIP-3-alpha expression. Noteworthy, MIP-3-beta levels were significantly higher in the pneumonia cohort over the entire course, starting from admission to the resuscitation room (day 0). Interleukins (IL-4, IL-6, IL-8, IL-10) and TNF-alpha expression were not associated with pneumonia, which underpins the high potential for MIP-3-alpha and -beta as novel biomarkers. Therefore, this pilot study may provide fundamental knowledge in the search for a precise biomarker for potential clinical use in future. Even though larger surveys need to be conducted to verify the present findings, this study is the first publication we know of demonstrating that patients suffering multiple traumas who have elevated MIP-3-alpha and MIP-3-beta levels are particularly vulnerable to pneumonia. Biomarkers that can accurately identify trauma patients predisposed to pneumonia would significantly enhance clinical practice, as their protein levels can be assessed at administration and objectively without the influence of subjective interpretation. Furthermore, the authors want to highlight that an AUC of 0.757 in MIP-3-alpha and MIP-3-beta is of limited clinical utility.

In the context of pneumonia and acute respiratory distress syndrome, the pulmonary resident cells are activated to secrete chemoattractants, thereby facilitating the migration of immune cells into the lung [23]. Chemokines are released due to bacterial components and initial inflammatory mediators, thereby creating a chemokine gradient that directs cells toward the site of inflammation [23]. MIP-3-alpha and MIP-3-beta are pro-inflammatory chemokines produced in the lung mucosa [19, 21]. These biomarkers are described in the development of pneumonia in SARS-CoV-2-infected patients [21]. Balnis et al. delineated that elevated concentrations of MIP-3-beta in the serum of patients suffering SARS-CoV-2-infection, who were mechanically ventilated, demonstrated a significant association with increased mortality rates and poor pulmonary outcomes [24]. In a similar study conducted by Chi et al. MIP-3-alpha levels were elevated in patients requiring mechanical ventilation [25]. In conclusion, elevated MIP-3-alpha and MIP-3-beta levels are associated with poor pulmonary outcomes as both chemokines promote inflammation. However, chemokines also seem to play a crucial role following trauma. Mukhametov et al. describe an increase of chemokines following trauma associated with purulent inflammatory complications [8]. After an initial rise of protein levels post-trauma, chemokines were documented to decrease continuously over time. In the prospective study, we saw a similar distribution of chemokine serum levels (MIP-3-alpha, MIP-3-beta) reaching their maximum on day 5, then continuously decreasing afterward. Our discovery of increased MIP-3-alpha levels and inflammation (pneumonia) post-trauma aligns with findings from one study Das et al. conducted [26]. They observed elevated MIP-3-alpha levels and active inflammation in the retina following TBI in a mice model seven-day post-trauma [26]. When CCR-6, the receptor of MIP-3-alpha, was knocked out, retinal pathologies and inflammation were significantly reduced, concluding that inflammation is associated with MIP-3-alpha expression following trauma. To our knowledge, there is no data correlating trauma and MIP-3-beta expression. Both MIP-3-alpha and MIP-3-beta can be secreted by cells of the innate immune system like neutrophils [27], macrophages [28], or DC [29], but also epithelial cells [30] in response to bacterial stimuli. Functionally, both chemokines regulate cellular migration [31–34], as well as the activation of DC, including maturation and antigen presentation [35, 36]. Additionally, MIP-3-alpha directly regulates bacterial dissemination in vivo by affecting cellular and humoral immunity and has antimicrobial properties [37, 38]. Given the association between the development of pneumonia and face- or head trauma in the present study, as also previously described by Zygun et al. [39], one might argue that pathogens that are present in the oral microbiome like *Staphylococcus aureus (S. aureus)* [40], a known cause of pneumonia in trauma patients, which was also the most prevalent pathogen in this study [2], might be disseminated and therefore lead to systemic elevation of MIP-3-alpha and -beta. Interestingly, peaks for both MIP-3-alpha and MIP-3-beta were observed 5 days after trauma, while pneumonia was diagnosed only by day 6.3 on average. This indicates that upon bacterial infection both cytokines are highly secreted and—among other cytokines—trigger a dysregulated immune response, which leads to the development of pneumonia [41]. Similar to pneumonia in SARS-CoV-2 patients, such dysregulated inflammation often precedes clinical diagnosis of pneumonia [42]. Therefore, the observed significant elevation of both cytokines before pneumonia diagnosis strengthens the potential as a biomarker for trauma-associated pneumonia. Furthermore, Interleukins such as IL-4, IL-6, IL-7, IL-8, IL-10, and TNF-alpha were not associated with pneumonia in the present study. This adheres with a recent report showing no significant difference in serum levels of cytokines usually related to systemic inflammation (e.g., IL-6) in trauma patients suffering from pneumonia compared to other trauma patients [43], which suggests a distinct functional role of these cytokines in trauma-associated pneumonia. Functional in vivo studies could be highly valuable in validating the chemokines as markers and evaluating the potential for therapeutic intervention targeting the chemokines.

## Limitations

Limitations of this study include a long period of inclusion. Furthermore, the study population was limited to a single level I trauma center, and a lack of blood samples, especially on day one post-trauma, was documented. As this is a pilot study, more extensive research is needed.

## Conclusion

Elevated MIP-3-alpha and MIP-3-beta levels are associated with pneumonia in patients suffering multiple traumas. The time courses from admission to day 10 show a notable increase followed by a decline with a peak of serum level expression on day 5 in both biomarkers. MIP-3-beta has been shown to be the more reliable biomarker, as its protein levels are significantly higher in the pneumonia cohort starting from the day of admission. In contrast, no comparable pattern was observed for other pro- and anti-inflammatory cytokines (IL-4, IL-6, IL-7, IL-8, IL-10, TNF-alpha). Therefore, we conclude that the present finding allows for identifying patients at high risk for developing pneumonia at an early stage. However, further basic research is crucial to elucidate the exact release mechanism of MIP-3-alpha and MIP-3-beta in trauma-associated pneumonia.

## Data Availability

The analyzed dataset in this study is available from the first author upon reasonable request. No datasets were generated or analyzed during the current study.
